# Supplementary material for: A Critical View of the Application of the APEX Software (Aqueous Photochemistry of Environmentally-Occurring Xenobiotics) to Predict Photoreaction Kinetics in Surface Freshwaters
Source: Molecules. 2019 Dec 18;25(1):9. doi: 10.3390/molecules25010009 (PMC7017383; doi:10.3390/molecules25010009)
Supplement: Supplementary file 1 [file molecules-25-00009-s001.zip › 3.2.4_gcc-4.4.0.zip!/3.2.4_gcc-4.4.0/3.2.4_gcc-4.4.0/doc/octave/html/interpreter/Overloading-Objects.html]

Overloading Objects - Untitled


Next: Inheritance and Aggregation,
Previous: Indexing Objects,
Up: Object Oriented Programming


---

### 33.4 Overloading Objects

- Function Overloading- Operator Overloading- Precedence of Objects
